# Dietary *N*-carbamylglutamate supplementation improves ammonia tolerance of juvenile yellow catfish *Pelteobagrus fulvidraco*

**DOI:** 10.3389/fphys.2023.1191468

**Published:** 2023-04-24

**Authors:** Dexiang Feng, Zhiguo Yang, Ming Li

**Affiliations:** ^1^ School of Fisheries, Xinyang Agriculture and Forestry University, Xinyang, China; ^2^ School of Marine Sciences, Ningbo University, Ningbo, China

**Keywords:** *N*-carbamylglutamate, ammonia, urea cycle, nitric oxide synthase, *Pelteobagrus fulvidraco*

## Abstract

**Introduction:** Ammonia has been of concern for its high toxicity to animals. *N*-carbamylglutamate (NCG) can reduce blood ammonia levels in mammals, but studies on ammonia tolerance in fish are insufficient.

**Methods:** Juvenile yellow catfish were fed two levels of NCG (0.00% and 0.05%) for 84 days under three ammonia levels (0.00, 0.08, and 0.16 mg/L NH_3_).

**Results and Discussion:** The results showed that survival rate (SUR), final body weight (FBW), weight gain (WG), and serum total protein (TP), triglycerides (TG), glucose (Glu), ornithine (Orn), citrulline (Cit) contents, and liver superoxide dismutase (SOD), catalase (CAT), glutathione peroxidase (GPx), arginase (ARG), ornithine transcarbamylase (OTC) activities decreased with the increase of ammonia levels, on the contrary, feed conversion ratio (FCR), hepatosomatic index (HSI), and serum ammonia, urea, alanine aminotransferase (ALT), aspartate aminotransferase (AST), glutamine (Gln), arginine (Arg) contents, and liver malondialdehyde (MDA), tumor necrosis factor (TNF), interleukin (IL) 1, IL 8 contents, and mRNA expressions of *cu/zn sod*, *cat*, *gpx*, *gr*, *tnf ɑ*, *il 1*, and *il 8* were significantly increased. Dietary 0.05% NCG supplementation had higher SUR, FBW, WG, feed intake (FI), whole-body protein, and serum TP, total cholesterol (TC), Glu, citrulline (Cit) contents, and liver SOD, GPx, argininosuccinate synthetase (ASS), argininosuccinate lyase (ASL), inducible nitric oxide synthase (iNOS) activities compared to 0.00% NCG group, but had lower serum ammonia, urea, ALT, AST, Gln, Arg contents, and liver MDA, TNF, IL 1, IL 8 contents, and neuronal nitric oxide synthase activity. At the end of bacterial challenge, cumulative mortality (CM) increased with ammonia levels increased, but serum antibody titer (AT), lysozyme (LYZ) activity, 50% hemolytic complement, immunoglobulin (Ig) contents, respiratory burst (RB), phagocytic indices decreased with ammonia levels increased. CM in 0.05% NCG group was lower than that in 0.00% NCG group, but serum AT, LYZ activity, Ig content, RB in 0.05% NCG group were significantly higher. The correlation analysis found that iNOS was positively correlated with ASS activity. This study indicates that dietary NCG supplementation can improve the ammonia tolerance of yellow catfish, and ASS may also be the target of NCG to activate the urea cycle.

## 1 Introduction

Ammonia is considered a toxic substance in aquaculture ecosystems, including unionized NH_3_ and NH_4_^+^ ions (Hegazi et al., 2010). The unionized NH_3_ causes poisoning in most fish, such as minnow *Rhynchocypris lagowski* (Yu et al., 2020), black sea bream *Acanthopagrus schlegelii* (Wang et al., 2020), common carp *Cyprinus carpio* (Xue et al., 2021), Nile tilapia *Oreochromis niloticus* (Esam et al., 2022), Japanese sea perch *Lateolabrax japonicus* (Zhang et al., 2022a) and hybrid snakehead *Channa maculatus* ♀ × *Channa argus* ♂ (Zuo et al., 2022). Acute or chronic ammonia stress can cause fish behavioral abnormalities, growth retardation, oxidative damage, and immunosuppression, making them more prone to disease outbreaks (Divya et al., 2020). It is well known that endogenous ammonia in fish is mostly excreted through gill tissues, but when ambient ammonia levels rise, they must convert it into less toxic substances, such as urea and glutamine (Ip and Chew, 2018). Recent studies have reported that improving the urea cycle efficiency of fish can effectively improve ammonia tolerance (Huang et al., 2019; Zhang et al., 2022b).

*N*-carbamylglutamate (NCG), an analogue of *N*-acetylglutamate (NAG), is a mandatory effector of the carbamyl phosphate synthase I (CPS I) reaction, an initial step in the urea cycle (Wu et al., 2004). In the feed industry, NCG has the advantages of being inexpensive and having a stable metabolism and high absorption rate compared with NAG (Chacher et al., 2013). NCG is clinically used to treat urea cycle disorder and restore ureagenesis and normalize blood ammonia levels in patients (Tuchman et al., 2008). In livestock and poultry breeding, NCG can significantly reduce their blood ammonia levels, increase plasma arginine contents, improve growth performance and antioxidant capacity, and inhibit inflammation, such in as chicken *Gallus gallus* (Huang et al., 2017), pig *Susscrofa domestica* (Wu et al., 2010; Yang et al., 2011), and sheep *Ovis aries* (Zhang et al., 2021). The vast majority of fish are known to excrete ammonia directly, so their urea cycle pathway may differ from that of mammals and birds (Anderson, 1995). In fact, many teleost species have enzymes involved in the urea cycle, such as mudskippers *Periophthalmodon schlosseri*, marble goby *Oxyeleotris marmoratus*, weather loach *Misgurnus anguillicaudatus*, small snakehead *Channa asiatica*, swamp eel *Monopterus albus*, mangrove killifish *Rivulus marmoratus*, central mud minnow *Umbra limi* (Ip and Chew, 2018), gulf toadfish *Opsanus beta*, oyster toadfish *Opsanus tau*, and plainfin midshipman *Porichthys notatus* (Wang and Walsh, 2000). So far, it has been reported that dietary NCG supplementation improves fish growth, but whether NCG can improve ammonia tolerance is unclear.

Yellow catfish is among the most economically valuable fish species in China, with a production of 587, 822 *t* in 2021 (MOAC, 2022). However, in recent years, ammonia stress has become the bottleneck in the development of yellow catfish aquaculture, and the yield and quality are being seriously degraded. The aim of this study was to investigate the changes in growth, blood health, antioxidant enzyme and ammonia metabolism enzyme activities, inflammation and disease resistance of yellow catfish fed a diet rich in NCG when ammonia stress occurs, so as to explore whether NCG can improve the ammonia tolerance of yellow catfish.

## 2 Materials and methods

### 2.1 Experimental diets

Two isonitrogenous (40% protein) and isolipidic (9% lipid) diets were prepared by adding 0.00% and 0.05% NCG to basal diets (Zhao et al., 2019). The NCG (97.5%) was supplied by Animore Sci. and Tech. Co., Ltd., Beijing, China. All the ingredients were ground into a powder through a 60-mesh, and the step-by-step expansion method was adopted to mix them evenly; then, they were processed into (2.00 × 2.00) mm pellets using a feed mill (F-26II, Science and Technology Industrial General Factory of South China University of Technology, China) and dried at room temperature to about 10% moisture. The diets were stored at −20°C until use. The diet formulation and proximate are presented in Table 1.

**TABLE 1:** The formula and approximate composition of the diets used in the experiment (% dry matter basis, DMB).

	0.00% NCG	0.05% NCG
Ingredients		
Fish meal	20.00	20.00
Soybean meal	22.00	22.00
Soy protein	16.00	16.00
Corn gluten meal	8.00	8.00
Cottonseed meal	1.50	1.50
Rapeseed meal	1.00	1.00
Fish oil	3.50	3.50
Soybean oil	3.50	3.50
Wheat flour	21.50	21.50
Vitamin premix^a^	0.50	0.50
Mineral premix^b^	0.50	0.50
Monocalcium phosphate	1.00	1.00
Alanine	0.05	0.00
*N*-carbamylglutamate	0.00	0.05
Proximate nutrition composition		
Protein	39.99	39.52
Lipid	8.89	8.95
*N*-carbamylglutamate	0.00	0.06

^a^ Vitamin premix (/kg diets): vitamin A, 5,500 IU; vitamin D_3_, 1,000 IU; vitamin K, 10 mg; niacin, 100 mg; riboflavin, 20 mg; pyridoxine, 20 mg; thiamin, 20 mg; biotin, 0.1 mg; D-calcium pantothenate, 50 mg; folacin, 5 mg; B_12_, 20 mg; ascorbic acid, 100 mg; inositol, 100 mg.

^b^ Mineral premix (mg/kg diets): NaCl, 500; MgSO_4_ · 7H_2_O, 4,575; NaH_2_PO_4_ · 2H_2_O, 12,500; KH_2_PO_4_, 16,000; Ca(H_2_PO_4_)_2_ · H_2_O, 6,850; FeSO_4_, 1,250; C_6_H_10_CaO_6_ · 5H_2_O, 1,750; ZnSO_4_ · 7H_2_O, 111; MnSO_4_ · 4H_2_O, 61.4; CuSO_4_ · 5H_2_O, 15.5; CoSO_4_ · 6H_2_O, 0.5; KI, 1.5.

### 2.2 Animal and experimental design

Juvenile yellow catfish were obtained from the Ningbo Aquatic Products Market, China. All fish were fed on a control experimental diet for 2 weeks. The fish (1.01 ± 0.02 g) were selected and randomly placed in 18,500 L cylindrical buckets (diameter of 0.8 m), with 60 fish per bucket.

Our previous study reported that the lethal concentration of 50% (LC50) of yellow catfish exposed to ammonia at 96 h was 57.00 mg/L total ammonia nitrogen (TA-N) (0.80 mg/L NH_3_; pH 6.6; 28°C) (Li et al., 2020). For the ammonia challenge, the fish were divided into a 0.00 mg/L NH_3_ group (<0.001 mg/L NH_3_; control group), 0.08 mg/L NH_3_ group (1/10 LC_50_), and 0.16 mg/L NH_3_ group (1/5 LC_50_). Ammonium chloride was added to the aquaculture system through a metering pump (Iwaki, Japan) to achieve the required ammonia levels. The actual ammonia levels were measured twice daily (YSI ProPlus Multi-Parameter Water Quality Instrument, YSI, United States). The fish were fed twice daily (07:00–07:30 a.m. and 18:00–18:30 p.m.) until apparent satiation for 84 days. The amounts of diet consumed were recorded daily. During the trail, the water temperature was 26°C–28°C, the pH was maintained within the range of 6.6–6.7, and the dissolved oxygen remained >7.00 mg/L and nitrate <0.1 mg/L, with a natural photoperiod.

### 2.3 Sample collections

We stopped feeding the experiment fish for 24 h, and then they were anesthetized (20 mg/L eugenol). Three fish from each bucket were randomly selected, minced, pooled, and stored at −20°C for a whole-body proximate composition analysis. We picked the other three fish from each bucket, and blood was collected from the tail vein, then centrifuged at 836 g to obtain serum and stored at −20°C for analysis of the serum biochemical index, free amino acid content, antioxidant enzyme activity, and inflammation response. Then, the liver samples were quickly removed and weighed for the hepatosomatic index, one part of which was stored at −20°C for ammonia metabolism enzyme activity analysis and another part of which was stored at −80°C for qRT-PCR analysis. All analyses were completed within 2 weeks of sampling.

### 2.4 Bacterial challenge

The frozen *Aeromonas hydrophila* was resuscitated in nutrient agar at 30°C under light for 48 h. The bacterial solution was concentrated to 1 × 10^8^ colony-forming units (CFU)/mL before the bacterial challenge.

After feeding for 84 days, 20 fish from each bucket were randomly selected and intraperitoneally injected with 0.1 mL of 1 × 10^6^ CFU/mL of *A. hydrophila* (Zhang et al., 2022b). Mortality was recorded daily during the 7 days of experiment, and dead fish were removed. We continued to feed all the fish experiment diets once a day, and the experimental conditions were maintained. At the end of the bacterial challenge, blood (three fish per bucket) was drawn from the caudal vasculature, and serum was stored at −20°C for analysis of the immune indexes; then, the head kidney was quickly removed for macrophage isolation.

### 2.5 Biochemical analysis

The diets and whole body of the fish were analyzed for proximate composition following the AOAC (2000) standard method. Protein was measured by the combustion method using the FP-528 Nitrogen Analyzer (Leco, United); lipid was measured by the ether extraction method using HT6 Soxtec System (FOSS, Sweden); ash was determined by incineration in the muffle furnace at 550°C for 8 h; and moisture was determined by oven drying at 105°C to a constant weight. The concentration of NCG was measured by high-performance liquid chromatography (HPLC; Agilent, California, United States).

A serum biochemical reagent (Nanjing Jiancheng Bioengineering Institute, Nanjing, China) was added to each well of the microtiter plate at 250 µL/well. Then, 5 µL of serum was added to each well. After 30-min incubation at room temperature, the analysis was carried out using the Hitachi 7600-110 automatic chemistry analyzer (Hitachi Ltd., Tokyo, Japan), including on the urea, total protein (TP), total cholesterol (TC), triglyceride (TG), glucose (Glu), alanine aminotransferase (ALT), and aspartate aminotransferase (AST). Serum ammonia content was detected by a kit (Nanjing Jiancheng, China) using a PT-3502C full-wavelength microplate reader (Beijing Potenov, China).

Serum was thoroughly mixed with 10 mmol/L D-nor-leucine and acetonitrile, and the supernatant was obtained by centrifugation. Serum free amino acid (glutamine, Gln; arginine, Arg; ornithine, Orn; citrulline, Cit) content was analyzed using a LC-20AD liquid chromatograph (Shumadzu, Japan).

Serum superoxide dismutase (SOD), catalase (CAT), and glutathione peroxidase (GPx) activities, and malondialdehyde (MDA) content were analyzed with commercially available assay kits (Nanjing Jiancheng Bioengineering Institute, Nanjing, China). One unit of SOD activity was calculated using the amount of superoxide dismutase required to inhibit the reduction of nitroblue tetrazolium by 50%; one unit of CAT activity was defined as the amount of CAT required to transform 1 μmol of H_2_O_2_ per min; and one unit of GPX activity was defined as the amount of GPX required to oxidize 1 μmol of NADPH per min.

Serum inflammation response (tumor necrosis factor, TNF; interleukin 1, IL 1; interleukin 8, IL 8), liver ammonia metabolism enzymes (argininosuccinate synthetase, ASS; argininosuccinate lyase, ASL; arginase, ARG; ornithine transcarbamylase, OTC), and nitric oxide synthase (neuronal nitric oxide synthase, nNOS; inducible nitric oxide synthase, iNOS) were determined by the ELISA method with kits (Nanjing Jiancheng, China). In short, TMB was converted to blue under the catalysis of peroxidase and finally to yellow under the action of acid. There was a positive correlation between the color and the cytokines in the samples. The absorbance was measured at 450 nm with a microplate reader, and the sample concentration was calculated.

Serum lysozyme (LYZ) activity was analyzed with a commercial assay kit (Nanjing Jiancheng Bioengineering Institute, Nanjing, China). The assay was based on the lysis of lysozyme-sensitive Gram-positive bacterium via the lysozyme present in the serum. Serum antibody titer (AT), 50% hemolytic complement (CH50), and total immunoglobulin (Ig) contents were analyzed using commercially available assay kits (Zhejiang Elikan Biological Technology Co., Ltd., Wenzhou, China). After 3 µL of serum was added to the 300 μL reagent, the sample was incubated for 10 min at 37°C. The absorbance of the samples was read at 340 nm. The head kidney was transferred to a L-15 culture medium (100 IU/mL penicillin, 100 μg/mL streptomycin, 10 IU/mL heparin, 2% fetal bovine serum), then filtered through a 100 μm metal mesh. The cell suspension was enriched by centrifugation at 600 g for 5 min at 4°C on the 34%/51% Percoll density gradient. The cells were collected at the 34%–51% interface and washed twice (cell concentration 1 × 10^7^/mL; cell viability >95%). The respiratory burst (RB) and phagocytic index (PI) were measured following the method of Zhang et al. (2018).

### 2.6 qRT-PCR analysis

Total RNA extraction was performed using the RNAiso Reagent kit (Takara, China), and cDNA was synthesized using the Prime Script PT reagent Kit (Takara, China). Table 2 lists the forward and reverse primers of genes. qRT-PCR was performed using a LightCycler^®^ 480 II Real-Time PCR system (Roche, Switzerland). The PCR temperature conditions were 95°C for 5 min, followed by 40 cycles of 95°C for 20 s, 57°C for 25 s, and 72°C for 25 s. Each sample was analyzed in triplicate, and the internal control genes included *β-actin* and *GAPDH*. The expression levels were calculated using the 2^−ΔΔCT^ method (Livak and Schmittgen, 2001).

**TABLE 2:** Primers used in this study.

Primer	Primer sequence (5′-3′)	Size (bp)
cu/zn sod	F: GGCGGAGATGATGAAAGT	105
	R: GAAAGGAAGCGGTGAAAC	
Cat	F: TCT​GTT​CCC​GTC​CTT​CAT​CC	151
	R: ATA​TCC​GTC​AGG​CAA​TCC​AC	
gpx	F: ATC​TAC​ATT​GGC​TTG​GAA​AC	257
	R: GAA​AGT​AGG​GAC​TGA​GGT​GA	
gr	F: CAG​TCG​CTT​TGT​TTG​TTC​TA	280
	R: TCC​TCC​GAT​ACA​CTT​CTC​AC	
tnf ɑ	F: AAC​CGA​AAG​GAA​GCA​CAG​AA	221
	R: TCA​CGG​CAA​TCG​TTT​AGG​AG	
il 1	F: TTG​AGA​AAC​GGA​CCC​GGT​GA	125
	R: AGG​TGG​CTG​GTT​TGC​TGA​TG	
il 8	F: CAA​GCC​AGC​AAT​GAC​CTC​T	227
	R: CAC​TGA​AGA​CAA​CCC​AAG​ACT	
β-actin	F: TTCGCTGGAGATGATGCT	136
	R: CGTGCTCAATGGGGTACT	
gapdh	F: TCT​GGG​GTA​CAC​AGA​ACA​CC	165
	R: ACT​AGG​TCA​CAG​ACA​CGG​TT	

### 2.7 Statistical analysis

All analyses were performed using SPSS 18.0.0 (SPSS, United States). Data were tested for normal distribution using the Kolmogorov–Smirnov test. The results were subjected to a two-way analysis of variance (ANOVA) followed by Tukey’s multiple range test. The correlations between the measured nitric oxide synthase activity and ammonia metabolism enzyme activity were conducted using the Pearson correlation. The level of significance was set at *p* < 0.05.

## 3 Results

### 3.1 Growth performance and body composition

The survival rate (SUR), final body weight (FBW), and weight gain (WG) decreased with the increase of ammonia levels (*p* < 0.05) (Table 3). On the contrary, the feed conversion ratio (FCR) and hepatosomatic index (HSI) were significantly increased (*p* < 0.05). SUR, FBW, WG, and feed intake (FI) in the 0.05% NCG group were significantly higher than those in the 0.00% NCG group (*p* < 0.05). The interactions of the dietary NCG supplementation and ammonia level were observed in SUR (*p* = 0.003), FBW (*p* = 0.048), and WG (*p* = 0.045).

**TABLE 3:** Effects of dietary *N*-carbamylglutamate supplementation on growth performance of yellow catfish exposed to different ammonia levels for 84 days.

NCG (%)	NH_3_ (mg/L)	SUR (g)	FBW (g)	WG	FI (%)	FCR (%)	HSI (%)
0.00	0.00	98.67 ± 1.15	23.52 ± 1.00	22.51 ± 1.01	21.33 ± 1.82	0.95 ± 0.04	1.78 ± 0.03
	0.08	93.33 ± 3.06	20.77 ± 0.67	19.76 ± 0.68	19.11 ± 0.81	0.97 ± 0.01	1.93 ± 0.05
	0.16	82.00 ± 2.00	19.94 ± 0.68	18.94 ± 0.68	20.00 ± 0.85	1.06 ± 0.06	1.93 ± 0.05
0.05	0.00	98.00 ± 2.00	27.12 ± 1.21	26.09 ± 1.22	24.27 ± 1.49	0.93 ± 0.03	1.70 ± 0.05
	0.08	97.33 ± 1.15	26.14 ± 0.85	25.14 ± 0.86	25.55 ± 0.67	1.02 ± 0.02	1.87 ± 0.02
	0.16	92.67 ± 3.06	24.56 ± 0.47	23.54 ± 0.45	24.40 ± 0.97	1.04 ± 0.03	1.87 ± 0.07
NCG level							
0.00		91.33 ± 7.62	21.41 ± 1.76	20.40 ± 1.76	20.15 ± 1.45	0.99 ± 0.06	1.88 ± 0.08
0.05		96.00 ± 3.16*	25.94 ± 1.36*	24.92 ± 1.36*	24.74 ± 1.13*	0.99 ± 0.05	1.82 ± 0.10
NH_3_ level							
0.00		98.33 ± 1.51^b^	25.32 ± 2.21^b^	24.30 ± 2.20^b^	22.80 ± 2.19	0.94 ± 0.03^a^	1.74 ± 0.06^a^
0.08		95.33 ± 3.01^b^	23.46 ± 3.02^a^	22.45 ± 3.03^a^	22.33 ± 3.59	0.99 ± 0.03^b^	1.90 ± 0.05^b^
0.16		87.33 ± 6.28^a^	22.25 ± 2.58^a^	21.24 ± 2.58^a^	22.20 ± 2.55	1.05 ± 0.04^c^	1.90 ± 0.06^b^
Two-way ANOVA							
NCG		0.049	0.001	0.001	0.001	0.874	0.150
NH_3_		0.001	0.016	0.016	0.928	0.001	0.001
NCG × NH_3_		0.003	0.048	0.045	0.069	0.191	0.883

Values are expressed as the mean ± SE (*n* = 3). The asterisks (*) indicate that they are significantly affected by NCG levels (*p* < 0.05). The different letters represent existing significant differences between the three ammonia levels (*p* < 0.05). Survival rate (SUR, %) = 100 × number of surviving fish/numbers of dead fish; Weight gain (WG, g) = final weight–initial weight; Feed conversion ratio (FCR) = dry diet fed (g)/wet weight gain (g); Hepatosomatic index (HSI, %) = 100 × liver weight (g)/body weight (g). NCG: *N*-carbamylglutamate; FBW (g): final body weight; FI (g): feed intake.

Whole-body protein content in the 0.05% NCG group was significantly higher than in the 0.00% NCG group (*p* < 0.05) (Table 4). Dietary NCG supplementation and ammonia stress had no effect on lipid, ash, and moisture contents (*p* > 0.05).

**TABLE 4:** Effects of dietary *N*-carbamylglutamate supplementation on whole-body proximate composition of yellow catfish exposed to different ammonia levels for 84 days.

NCG (%)	NH_3_ (mg/L)	Protein (% DMB)	Lipid (% DMB)	Ash (% DMB)	Moisture (%)
0.00	0.00	18.45 ± 0.86	11.64 ± 0.55	40.98 ± 0.09	66.86 ± 0.44
	0.08	18.66 ± 0.68	11.59 ± 0.47	41.18 ± 0.50	67.46 ± 0.98
	0.16	18.78 ± 0.42	12.29 ± 0.43	40.92 ± 0.43	66.49 ± 1.69
0.05	0.00	21.69 ± 0.50	11.96 ± 0.99	40.90 ± 0.68	65.98 ± 0.77
	0.08	21.50 ± 0.82	11.80 ± 0.31	40.81 ± 0.77	66.55 ± 0.94
	0.16	20.76 ± 0.33	11.78 ± 0.46	40.79 ± 0.19	67.52 ± 0.52
NCG level					
0.00		18.63 ± 0.60	11.84 ± 0.54	41.03 ± 0.35	66.82 ± 0.97
0.05		21.28 ± 0.65*	11.85 ± 0.57	40.83 ± 0.53	66.68 ± 0.94
NH_3_ level					
0.00		20.07 ± 1.88	11.80 ± 0.74	40.94 ± 0.44	66.42 ± 0.74
0.08		20.03 ± 1.64	11.70 ± 0.37	41.00 ± 0.62	67.00 ± 0.99
0.16		18.77 ± 1.13	12.04 ± 0.49	40.86 ± 0.31	66.84 ± 1.10
Two-way ANOVA					
NCG		0.001	0.973	0.366	0.760
NH_3_		0.938	0.564	0.873	0.569
NCG × NH_3_		0.260	0.423	0.869	0.058

Values are expressed as the mean ± SE (*n* = 3). The asterisk (*) indicates that they are significantly affected by NCG levels (*p* < 0.05).

### 3.2 Serum biochemical index and free amino acid content

The serum ammonia, urea, ALT, and AST contents increased as ammonia levels increased, while TP, TG, and Glu contents decreased (*p* < 0.05) (Table 5). Serum ammonia, urea, ALT, and AST in the 0.05% NCG group were significantly lower than those in the 0.00% NCG group, but TP, TC, and Glu contents were significantly higher than those in the 0.00% NCG group (*p* < 0.05). The interactions of the dietary NCG supplementation and ammonia level were observed in serum ammonia (*p* = 0.001), urea (*p* = 0.001), TP (*p* = 0.014), Glu (*p* = 0.001), ALT (*p* = 0.001), and AST (*p* = 0.001).

**TABLE 5:** Effects of dietary *N*-carbamylglutamate supplementation on serum biochemical index of yellow catfish exposed to different ammonia levels for 84 days.

NCG (%)	NH_3_ (mg/L)	Ammonia (µmol/L)	Urea (mmol/L)	TP (g/L)	TC (mmol/L)	TG (mmol/L)	Glu (mmol/L)	ALT (mg/L)	AST (mg/L)
0.00	0.00	292.98 ± 5.67	7.30 ± 0.33	27.94 ± 0.69	7.18 ± 0.05	5.82 ± 0.19	6.76 ± 0.10	9.74 ± 0.96	22.33 ± 1.23
	0.08	568.31 ± 20.26	25.97 ± 0.99	25.23 ± 1.01	7.35 ± 0.16	5.44 ± 0.08	5.17 ± 0.07	27.12 ± 1.72	32.25 ± 1.33
	0.16	937.85 ± 27.26	28.39 ± 0.96	23.45 ± 1.07	7.34 ± 0.09	5.19 ± 0.06	4.49 ± 0.17	30.81 ± 1.79	36.63 ± 0.60
0.05	0.00	292.55 ± 4.60	5.65 ± 0.38	29.01 ± 0.73	7.46 ± 0.14	5.74 ± 0.13	6.93 ± 0.17	5.70 ± 0.60	18.44 ± 0.74
	0.08	337.09 ± 4.05	12.63 ± 0.86	27.89 ± 0.11	7.47 ± 0.19	5.41 ± 0.18	6.20 ± 0.11	10.49 ± 1.69	24.80 ± 1.11
	0.16	343.28 ± 10.97	18.60 ± 0.87	27.57 ± 0.38	7.55 ± 0.09	5.15 ± 0.09	5.32 ± 0.13	12.67 ± 2.12	26.30 ± 1.24
NCG level									
0.00		599.71 ± 80.76	20.56 ± 5.02	25.54 ± 2.12	7.29 ± 0.13	5.49 ± 0.30	5.47 ± 1.02	22.55 ± 9.83	30.40 ± 6.41
0.05		324.31 ± 24.78*	12.29 ± 5.65*	28.16 ± 0.78*	7.50 ± 0.13*	5.43 ± 0.28	6.15 ± 0.71*	9.62 ± 3.38*	23.18 ± 3.73*
NH_3_ level									
0.00		292.77 ± 4.63^a^	6.48 ± 0.96^a^	28.48 ± 0.87^b^	7.32 ± 0.18	5.78 ± 0.15^c^	6.85 ± 0.16^c^	7.72 ± 2.32^a^	20.39 ± 2.32^a^
0.08		452.70 ± 27.31^b^	19.30 ± 3.35^b^	26.56 ± 1.59^a^	7.41 ± 0.17	5.43 ± 0.12^b^	5.68 ± 0.57^b^	18.81 ± 3.23^b^	28.52 ± 4.22^b^
0.16		640.57 ± 26.19^c^	23.50 ± 5.43^c^	25.51 ± 2.37^a^	7.45 ± 0.14	5.17 ± 0.07^a^	4.90 ± 0.47^a^	21.74 ± 5.09^c^	31.47 ± 5.72^c^
Two-way ANOVA									
NCG		0.010	0.047	0.003	0.004	0.694	0.021	0.002	0.010
NH_3_		0.030	0.001	0.028	0.410	0.001	0.001	0.020	0.021
NCG × NH_3_		0.001	0.001	0.014	0.585	0.934	0.001	0.001	0.001

Values are expressed as the mean ± SE (*n* = 3). The asterisks (*) indicate that they are significantly affected by NCG levels (*p* < 0.05). The different letters represent existing significant differences between the three ammonia levels (*p* < 0.05). TP; total protein, TC; total cholesterol, TG; triglycerides, Glu; glucose, ALT; alanine aminotransferase, AST; aspartate aminotransferase.

The serum Gln and Arg contents increased as ammonia levels increased, while the Orn and Cit contents decreased (*p* < 0.05) (Table 6). The serum Gln and Arg contents in the 0.05% NCG group were significantly lower than those in the 0.00% NCG group, but the Cit content was higher (*p* < 0.05). The interactions of the dietary NCG supplementation and ammonia level were observed in Gln (*p* = 0.001), Arg (*p* = 0.001), and Cit (*p* = 0.009).

**TABLE 6:** Effects of dietary *N*-carbamylglutamate supplementation on serum free amino acid content of yellow catfish exposed to different ammonia levels for 84 days.

NCG (%)	NH_3_ (mg/L)	Gln (µg/L)	Arg (µg/L)	Orn (µg/L)	Cit (µg/L)
0.00	0.00	49.65 ± 1.22	11.79 ± 0.98	322.48 ± 8.72	258.68 ± 7.34
	0.08	53.04 ± 1.54	13.53 ± 0.51	306.88 ± 5.18	214.67 ± 7.34
	0.16	66.93 ± 2.87	15.37 ± 0.62	308.91 ± 3.27	179.12 ± 8.14
0.05	0.00	53.34 ± 1.99	11.94 ± 0.81	319.71 ± 6.75	259.95 ± 9.60
	0.08	84.61 ± 3.62	15.88 ± 0.19	308.38 ± 7.03	214.19 ± 11.40
	0.16	97.15 ± 2.49	23.09 ± 0.92	305.19 ± 4.01	213.15 ± 10.11
NCG level					
0.00		78.36 ± 9.69	16.97 ± 4.94	312.76 ± 9.07	217.49 ± 35.07
0.05		56.54 ± 8.12*	13.56 ± 1.68*	311.10 ± 8.45	229.10 ± 24.83*
NH_3_ level					
0.00		51.50 ± 2.50^a^	11.86 ± 0.81^a^	321.10 ± 7.14^b^	259.31 ± 7.68^c^
0.08		68.82 ± 7.47^b^	14.71 ± 1.33^b^	307.63 ± 5.58^a^	214.43 ± 8.14^b^
0.16		82.04 ± 6.73^c^	19.23 ± 4.29^c^	307.05 ± 3.85^a^	196.13 ± 20.37^a^
Two-way ANOVA					
NCG		0.007	0.047	0.693	0.043
NH_3_		0.007	0.001	0.001	0.001
NCG × NH_3_		0.001	0.001	0.739	0.009

Values are expressed as the mean ± SE (*n* = 3). The asterisks (*) indicate that they are significantly affected by NCG levels (*p* < 0.05). The different letters represent existing significant differences between the three ammonia levels (*p* < 0.05). Gln; glutamine, Arg; arginine, Orn; ornithine, Cit; citrulline.

### 3.3 Antioxidant enzyme activity and inflammation

Serum SOD, CAT, and GPx activities decreased as ammonia levels increased, while MDA, TNF, IL 1, and IL 8 contents decreased (*p* < 0.05) (Table 7). Serum SOD and GPx activities in the 0.05% NCG group were significantly higher than those in the 0.00% NCG group, but MDA, TNF, IL 1, and IL 8 contents were lower (*p* < 0.05). The interactions of the dietary NCG supplementation and ammonia level were observed in SOD (*p* = 0.020), CAT (*p* = 0.016), GPx (*p* = 0.001), MDA (*p* = 0.025), TNF (*p* = 0.001), IL 1(*p* = 0.001), and IL 8 (*p* = 0.017).

**TABLE 7:** Effects of dietary *N*-carbamylglutamate supplementation on serum antioxidant enzyme activity and inflammation of yellow catfish exposed to different ammonia levels for 84 days.

NCG (%)	NH_3_ (mg/L)	SOD (U/mL)	CAT (U/mL)	GPx (U/mL)	MDA (nmol/mL)	TNF (pg/mL)	IL1 (pg/mL)	IL8 (pg/mL)
0.00	0.00	123.67 ± 1.44	30.25 ± 1.32	318.74 ± 3.15	1.64 ± 0.06	821.59 ± 12.33	3,165.37 ± 81.10	911.88 ± 9.86
	0.08	108.30 ± 3.37	26.74 ± 1.28	298.55 ± 3.41	1.92 ± 0.06	1,075.01 ± 43.23	3,905.30 ± 9.00	1,157.52 ± 39.57
	0.16	97.26 ± 5.25	26.37 ± 1.80	282.37 ± 2.79	2.32 ± 0.09	1,244.02 ± 8.17	4,291.89 ± 37.55	1,345.49 ± 21.77
0.05	0.00	132.59 ± 2.52	30.39 ± 0.97	327.37 ± 3.15	1.52 ± 0.03	806.61 ± 5.11	3,037.48 ± 53.24	908.39 ± 5.84
	0.08	125.33 ± 3.35	28.93 ± 2.47	325.15 ± 5.68	1.68 ± 0.03	812.86 ± 8.09	3,095.78 ± 36.19	937.62 ± 13.06
	0.16	102.12 ± 2.88	28.42 ± 1.06	312.00 ± 8.02	1.98 ± 0.07	939.22 ± 23.28	3,272.95 ± 20.02	1,148.48 ± 139.64
NCG level								
0.00		109.74 ± 1.92	27.79 ± 2.26	299.89 ± 16.01	1.96 ± 0.30	1,046.87 ± 85.54	3,787.52 ± 97.76	1,138.30 ± 89.73
0.05		120.01 ± 4.02*	27.24 ± 3.94	321.51 ± 8.02*	1.73 ± 0.21*	852.90 ± 66.01*	3,135.40 ± 11.43*	998.16 ± 33.40*
NH_3_ level								
0.00		128.12 ± 5.22^c^	30.32 ± 1.04^c^	323.06 ± 5.51^c^	1.58 ± 0.08^a^	814.10 ± 11.77^a^	3,101.42 ± 93.12^a^	910.13 ± 7.50^a^
0.08		116.82 ± 9.80^b^	27.83 ± 2.13^b^	311.84 ± 15.16^b^	1.80 ± 0.13^b^	943.94 ± 46.25^b^	3,500.54 ± 44.02^b^	1,047.57 ± 23.29^b^
0.16		99.69 ± 4.63^a^	24.40 ± 2.53^a^	297.18 ± 16.42^a^	2.15 ± 0.20^c^	1,091.62 ± 67.67^c^	3,782.42 ± 58.75^c^	1,246.99 ± 40.12^c^
Two-way ANOVA								
NCG		0.013	0.724	0.002	0.043	0.005	0.001	0.020
NH_3_		0.001	0.001	0.014	0.001	0.007	0.039	0.001
NCG × NH_3_		0.020	0.016	0.001	0.025	0.001	0.001	0.017

Values are expressed as the mean ± SE (*n* = 3). The asterisks (*) indicate that they are significantly affected by NCG levels (*p* < 0.05). The different letters represent existing significant differences between the three ammonia levels (*p* < 0.05). SOD; superoxide dismutase, CAT; catalase, GPx; glutathione peroxidase, MDA; malondialdehyde, TNF; tumor necrosis factor, IL 1; interleukin 1, IL 8, interleukin 8.

**FIGURE 1:**
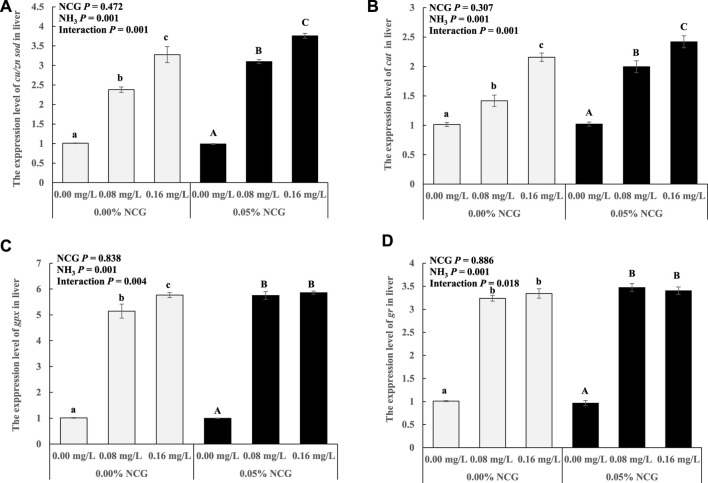
Relative expression levels of antioxidant-related genes *cu/zn sod* **(A)**, *cat* **(B)**, *gpx* **(C)**, and *gr* **(D)** in liver. The relative expression of the transcript from qRT-PCR was calculated based on the standard curve and normalized to the *β-actin* and *gapdh* mRNA level (*n* = 3). The different letters represent existing significant differences between the three ammonia levels (*p* < 0.05).

The *cu/zn sod*, *cat*, *gpx*, and *gr* expressions in the liver increased as ammonia levels increased (*p* < 0.05) (Figure 1). The interactions of the dietary NCG supplementation and ammonia level were observed in *cu/zn sod* (*p* = 0.001), *cat* (*p* = 0.001), *gpx* (*p* = 0.004), and *gr* (*p* = 0.018) expressions.

**FIGURE 2:**
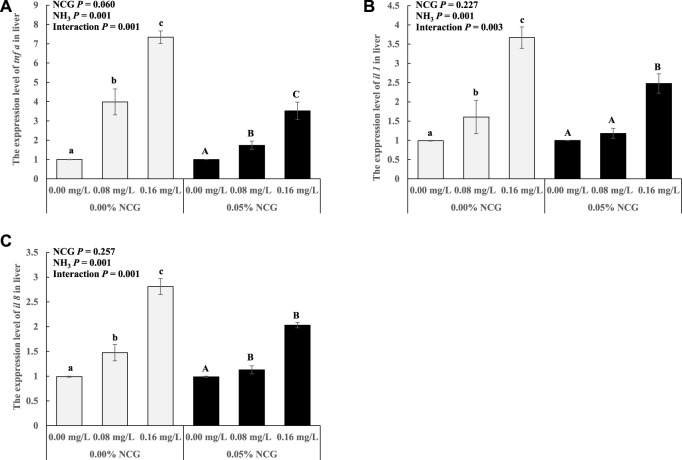
Relative expression levels of antioxidant-related genes *tnf ɑ* **(A)**, *il 1* **(B)**, and *il 8* **(C)** in liver. The relative expression of the transcript from qRT-PCR was calculated based on the standard curve and normalized to the *β-actin* and *gapdh* mRNA level (*n* = 3). The different letters represent existing significant differences between the three ammonia levels (*p* < 0.05).

The *tnf ɑ*, *il 1,* and *il 8* expression in the liver increased as ammonia levels increased (*p* < 0.05) (Figure 2). The interactions of the dietary NCG supplementation and ammonia level were observed in *tnf ɑ* (*p* = 0.001), *il 1* (*p* = 0.003), and *il 8* (*p* = 0.001) expression.

### 3.4 Ammonia metabolism enzyme activity

The liver ARG and OTC activities decreased as ammonia levels increased (*p* < 0.05) (Table 8). The liver ASS, ASL, and iNOS activities in the 0.05% NCG group were significantly higher than those in the 0.00% NCG group, but nNOS activity was lower (*p* < 0.05). The interactions of the dietary NCG supplementation and ammonia level were observed in ASS (*p* = 0.001), ASL (*p* = 0.001), nNOS (*p* = 0.001), and iNOS (*p* = 0.001).

**TABLE 8:** Effects of dietary *N*-carbamylglutamate supplementation on activities of ammonia metabolism enzyme and nitric oxide synthase in liver of yellow catfish exposed to different ammonia levels for 84 days.

NCG (%)	NH_3_ (mg/L)	ASS (U/mg protein)	ASL (U/mg protein)	ARG (U/mg protein)	OTC (U/mg protein)	nNOS (U/mg protein)	iNOS (U/mg protein)
0.00	0.00	944.51 ± 10.69	2,178.28 ± 69.67	1,532.52 ± 17.29	996.28 ± 10.48^c^	1.63 ± 0.05	0.71 ± 0.07
	0.08	842.77 ± 11.38	2057.04 ± 52.17	1,267.58 ± 52.91	865.25 ± 15.96	1.81 ± 0.08	0.56 ± 0.06
	0.16	771.35 ± 13.50	1806.38 ± 32.11	976.12 ± 6.58	741.58 ± 26.81	1.91 ± 0.04	0.47 ± 0.03
0.05	0.00	960.70 ± 7.09	2,178.28 ± 69.67	1,611.77 ± 20.37	1,072.07 ± 57.91	1.39 ± 0.05	0.88 ± 0.03
	0.08	981.08 ± 17.52	2,372.72 ± 70.10	1,397.74 ± 13.96	972.52 ± 44.34	1.21 ± 0.07	0.99 ± 0.04
	0.16	1,120.68 ± 53.36	2,352.38 ± 85.23	1,111.06 ± 104.56	853.01 ± 61.92	1.23 ± 0.02	1.16 ± 0.08
NCG level							
0.00		852.88 ± 76.06	2013.90 ± 70.68	1,258.76 ± 42.67	867.70 ± 10.52	1.78 ± 0.13	0.58 ± 0.12
0.05		1,020.86 ± 80.52*	2,291.57 ± 23.71*	1,373.52 ± 24.10	965.87 ± 16.35	1.27 ± 0.10*	1.10 ± 0.13*
NH_3_ level							
0.00		952.66 ± 12.06	2,163.95 ± 59.51	1,572.18 ± 45.55^c^	1,043.17 ± 55.76^c^	1.51 ± 0.14	0.80 ± 0.10
0.08		911.92 ± 6.90	2,214.88 ± 181.52	1,332.66 ± 79.25^b^	918.89 ± 65.88^b^	1.51 ± 0.33	0.78 ± 0.24
0.16		946.02 ± 94.48	2079.38 ± 304.55	1,043.59 ± 99.26^a^	797.30 ± 74.47^a^	1.57 ± 0.38	0.82 ± 0.39
Two-way ANOVA							
NCG		0.001	0.001	0.313	0.074	0.001	0.001
NH_3_		0.824	0.535	0.001	0.001	0.920	0.970
NCG × NH_3_		0.001	0.001	0.572	0.723	0.001	0.001

Values are expressed as the mean ± SE (*n* = 3). The asterisks (*) indicate that they are significantly affected by NCG levels (*p* < 0.05). The different letters represent existing significant differences between the three ammonia levels (*p* < 0.05). ASS; argininosuccinate synthetase, ASL; argininosuccinate lyase, ARG; arginase, OTC; ornithine transcarbamylase, nNOS; neuronal nitric oxide synthase, iNOS; inducible nitric oxide synthase.

### 3.5 Bacterial challenge

At the end of the bacterial challenge, CM increased as ammonia levels increased (*p* < 0.05) (Table 9). The serum AT and LYZ activity, the CH50 and Ig content, and RB and PI decreased as ammonia levels increased (*p* < 0.05). CM in the 0.05% NCG group was lower than in the 0.00% NCG group, but serum AT and LYZ activity, Ig content, and RB in the 0.05% NCG group were significantly higher than in the 0.00% NCG group (*p* < 0.05). The interactions of the dietary NCG supplementation and ammonia level were observed in CM (*p* = 0.021), LYZ (*p* = 0.001), Ig (*p* = 0.001), RB (*p* = 0.001), and PI (*p* = 0.007).

**TABLE 9:** Effects of dietary *N*-carbamylglutamate supplementation and ammonia stress on cumulative mortality and serum immune response of yellow catfish 7 days post-challenge with *A. hydrophila*.

NCG (%)	NH_3_ (mg/L)	CM (%)	AT (log_10_)	LYZ (U/mL)	CH50 (mg/mL)	Ig (mg/mL)	RB	PI (%)
0.00	0.00	31.67 ± 7.64	1.97 ± 0.02	85.03 ± 3.94	64.57 ± 1.45	20.53 ± 0.43	1.89 ± 0.13	1.02 ± 0.07
	0.08	46.67 ± 7.64	1.57 ± 0.03	71.82 ± 1.34	58.46 ± 1.93	15.30 ± 0.92	1.49 ± 0.05	0.71 ± 0.03
	0.16	80.00 ± 5.00	1.48 ± 0.05	62.61 ± 3.34	54.23 ± 0.96	11.15 ± 0.75	1.14 ± 0.12	0.54 ± 0.04
0.05	0.00	18.33 ± 5.77	2.21 ± 0.02	86.06 ± 2.21	64.10 ± 1.48	20.89 ± 0.50	1.95 ± 0.07	1.02 ± 0.03
	0.08	38.33 ± 7.64	1.84 ± 0.03	82.45 ± 1.15	59.05 ± 1.03	18.12 ± 0.44	1.71 ± 0.02	0.83 ± 0.02
	0.16	46.67 ± 7.64	1.82 ± 0.03	80.74 ± 0.44	57.36 ± 0.96	16.71 ± 0.36	1.66 ± 0.05	0.71 ± 0.03
NCG level								
0.00		52.78 ± 22.24	1.67 ± 0.23	73.15 ± 5.12	58.09 ± 4.68	15.66 ± 4.12	1.51 ± 0.34	0.76 ± 0.21
0.05		34.44 ± 14.02*	1.96 ± 0.19*	83.08 ± 2.67*	60.17 ± 3.20	18.57 ± 1.88*	1.77 ± 0.14*	0.85 ± 0.13
NH_3_ level								
0.00		25.00 ± 9.49^a^	2.09 ± 0.14^b^	85.54 ± 2.91^b^	64.33 ± 1.34^c^	20.71 ± 0.46^c^	1.92 ± 0.09^c^	1.02 ± 0.05^c^
0.08		42.50 ± 8.22^b^	1.71 ± 0.15^b^	77.14 ± 5.92^a^	58.76 ± 1.42^b^	16.70 ± 1.67^b^	1.60 ± 0.12^b^	0.77 ± 0.07^b^
0.16		63.33 ± 19.15^c^	1.65 ± 0.19^a^	71.68 ± 10.16^a^	55.80 ± 1.92^a^	13.93 ± 3.09^a^	1.40 ± 0.30^a^	0.63 ± 0.10^a^
Two-way ANOVA								
NCG		0.043	0.011	0.012	0.575	0.042	0.045	0.270
NH_3_		0.001	0.001	0.012	0.001	0.001	0.001	0.001
NCG × NH_3_		0.021	0.053	0.001	0.098	0.001	0.001	0.007

Values are expressed as the mean ± SE (*n* = 3). The asterisks (*) indicate that they are significantly affected by NCG levels (*p* < 0.05). The different letters represent existing significant differences between the three ammonia levels (*p* < 0.05). CM; cumulative mortality, AT; antibody titer, LYZ; lysozyme, CH50; 50% hemolytic complement, Ig; immunoglobulin, RB; respiratory burst, PI; phagocytic indices.

### 3.6 Correlation analysis

**FIGURE 3:**
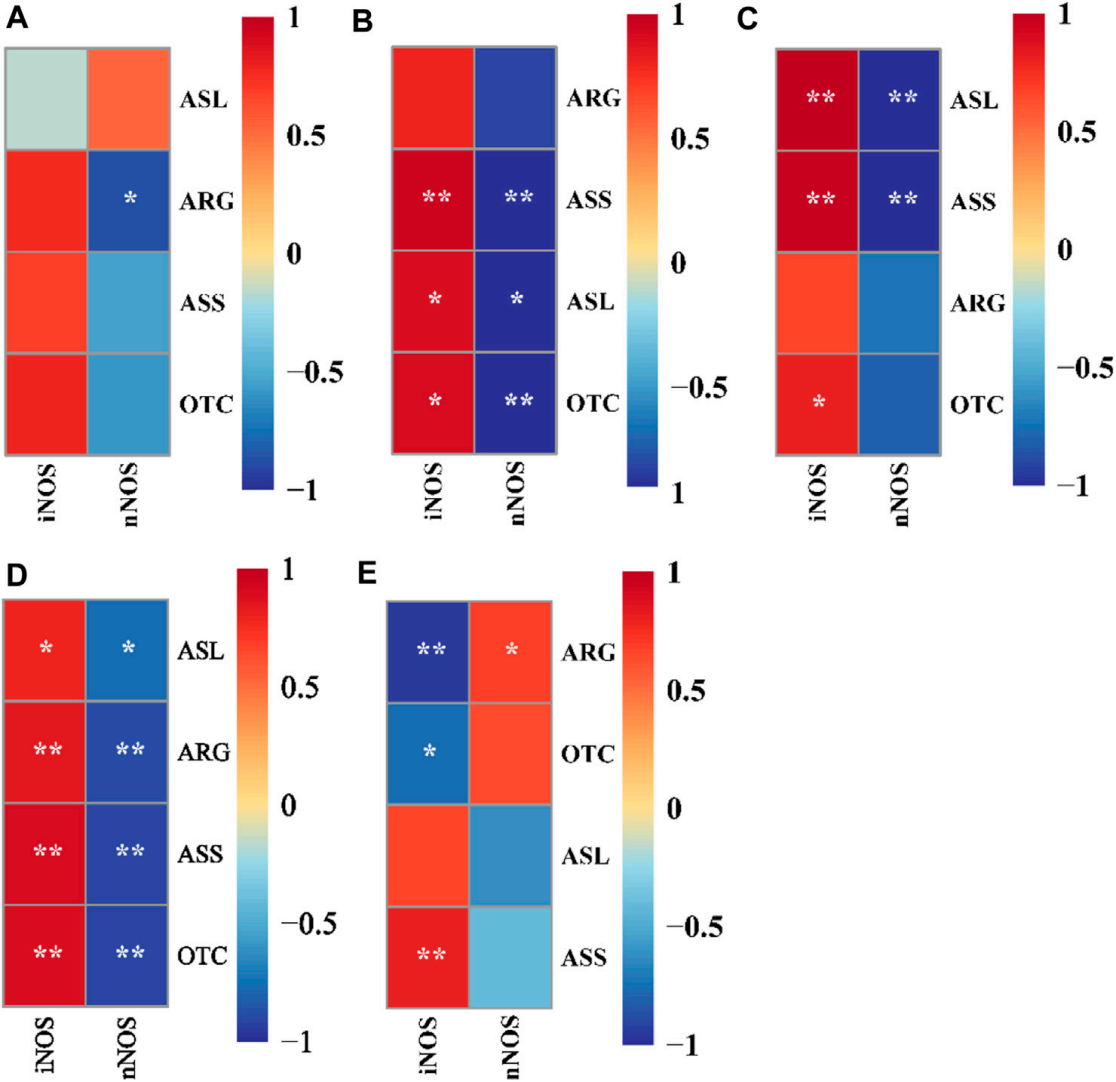
Correlation analysis between nitric oxide synthase activity and ammonia metabolism enzyme activity in yellow catfish fed with different N-carbamylglutamate under 0.00 mg/L NH3 **(A)**, 0.08 mg/L NH3 **(B)**, and 0.16 mg/L NH3 **(C)** ammonia levels, respectively. Correlation analysis between nitric oxide synthase activity and ammonia metabolism enzyme activity in yellow catfish fed diets with 0.00% **(D)** and 0.05% **(E)** N-carbamylglutamate under different ammonia levels. A significant difference was marked as ∗ at *p* < 0.05 and ∗∗ at *p* < 0.001. nNOS; neuronal nitric oxide synthase, iNOS; inducible nitric oxide synthase, ASS; argininosuccinate synthetase, ASL; argininosuccinate lyase, ARG; arginase, OTC; ornithine transcarbamylase.

As shown in Figure 3, nNOS was significantly negatively correlated with ARG in 0.00 mg/L NH_3_ level (*p* < 0.05). iNOS was significantly positively correlated with ASS, ASL, and OTC in 0.16 mg/L NH_3_ level, but nNOS was significantly negatively correlated with ASS, ASL, and OTC (*p* < 0.05). iNOS was significantly positively correlated with ASL, ASS, and OTC in 0.16 mg/L NH_3_ level, but nNOS was significantly negatively correlated with ASL and ASS (*p* < 0.05). Dietary 0.00% NCG supplementation and iNOS were significantly positively correlated with ASL, ARG, ASS, and OTC, but those trends were reversed in nNOS (*p* < 0.05). Dietary 0.05% NCG supplementation and iNOS were significantly positively correlated with ASS but significantly negatively correlated with ARG and OTC, and nNOS was positively correlated with ARG (*p* < 0.05).

## 4 Discussion

To protect fishery resources, the Chinese government has recommended a threshold of 0.020 mg/L NH_3_ for fisheries (Fan et al., 2021). However, the NH_3_ levels in the intensive aquaculture systems are usually maintained at 0.052–0.064 mg/L (data not published), which is more than upper limit of ammonia tolerance in many cultured fishes, such as burbot *Lota lota*, Atlantic salmon *Salmo salar*, Pacific cod *Gadus macrocephalus*, rainbow trout, channel catfish *Ictalurus punctatus* (Vaage and Myrick, 2021), golden pompano *Trachinotus ovatus* (Liu et al., 2021), Japanese sea perch (Zhang et al., 2022a), and Nile tilapia (Esam et al., 2022). Similarly, this study also found that ammonia stress (>0.16 mg/L NH_3_) can lead to a lower survival rate of yellow catfish. It is worth noting that the dietary 0.05% NCG supplementation significantly increased the survival rate of yellow catfish at this ammonia level, which may be related to the reduction of stress, energy consumption, and internal ammonia load. A previous study confirmed that dietary NCG contents at 0.03%–0.05% can improve the growth, digestive enzyme activity, oxidation resistance status, and immunity of yellow catfish (Zhao et al., 2019). As was expected, we found that the dietary 0.05% NCG supplementation improves the growth (FBW and WG) of yellow catfish under ammonia stress.

Fish mainly rely on gill tissue to remove toxic ammonia from their bodies, but when the ambient ammonia is too high, some fish also can convert ammonia to non-toxic urea by the urea cycle pathway, such as marble goby (Jow et al., 1999), mudskippers (Lim et al., 2001), weather loach (Chew et al., 2001), swamp eel (Tay et al., 2003), walking catfish *Clarias batrachus* (Banerjee et al., 2017), magur catfish *Clarias magur* (Banerjee et al., 2020), common carp (Xue et al., 2021), and rainbow trout (Clark et al., 2019). The urea cycle consists of five key enzymes, namely, CPS I, ornithine transcarbamylase (OTC), argininosuccinate synthetase (ASS), argininosuccinate lyase (ASL), and arginase (ARG). Recent studies have suggested that ARG deficiency may be a major cause of ammonia poisoning in some fish, such as rainbow trout (Clark et al., 2019), Dolly Varden char *Salvelinus malma* (Zhu et al., 2020), and yellow catfish (Zhang et al., 2022b). NCG is mainly used to treat urea cycle disorders caused by CPS I deficiency in clinical practice (Ucar et al., 2009). Although this study found that dietary NCG could not alleviate the inhibition of ARG activity caused by ammonia stress, serum Arg and urea accumulations were alleviated, which indicates that dietary NCG can alleviate urea cycle disorder in fish to a certain extent. In addition, this study found that dietary NCG increased the activities of ASS and ASL, which are key enzymes linking the urea and nitric oxide synthesis (Husson et al., 2003). Nitric oxide is considered to be a very important immune signaling molecule (Colasanti and Suzuki, 2000). Unlike mammals, nitric oxide in fish is produced by two nitric oxide synthase isoforms: inducible nitric oxide synthase (iNOS) and neuronal nitric oxide synthase (nNOS) (Vemuganti and Raghavendra, 2002). nNOS exists in the central nervous system (CNS), and its production of nitric oxide can affect the neuron function in the brain, which is considered the main mediator of neuronal death (Kiss, 2000). A previous study reported that nNOS knockout mice were more resistant to neuro-excitotoxic injury (Ayata et al., 1997). Fish ammonia poisoning typically results in abnormal behavior, including polypnea, hyperexcitability, mania, convulsions, and syncope (Zhang et al., 2022b). In this study, an important finding was that dietary 0.05% NCG supplementation decreased nNOS activity, which proves that NCG could alleviate the neurotoxicity caused by ammonia poisoning in yellow catfish. In addition, 0.05% NCG was added to the diet, which significantly increased iNOS activity in the liver of yellow catfish. iNOS was isolated from macrophages and involved in mitochondrial superoxide anion scavenging (Moncada and Higgs, 1993), and it mediated immune functions (Iadecola, 1997). Based on this finding, we hypothesized that NCG may have a positive effect on the health status of yellow catfish under ammonia stress, including immunity and disease resistance.

In this study, ammonia stress was found to induce the deterioration of blood health (TP, TG, and Glu decreased), which is consistent with other ammonia poisoning fish, such as blunt snout bream *Megalobrama amblycephala* (Zhang et al., 2019), Dolly Varden char (Zhu et al., 2020), and common carp (Xue et al., 2022). A recent study reported that dietary NCG supplements effectively improved the blood health of Japanese seabass, decreasing plasma low-density lipoproteins and ammonia contents, increasing antioxidant enzyme activity, and decreasing inflammation and apoptosis (Huang et al., 2019). This is consistent with the findings of this study, in which dietary 0.05% NCG supplementation decreased serum ammonia and urea contents and increased TP, TC, and Glu contents. In addition, NCG has also been reported to improve liver health in animals (Huang et al., 2019). The AST and ALT are indicators that can be released into the bloodstream following the occurrence of liver damage (Lin et al., 2010). This study found that dietary 0.05% NCG supplementation decreased the AST and ALT contents in the serum of yellow catfish. This may be related to the antioxidant capacity of NCG, which reduces oxidative damage of the liver induced by ammonia toxicity.

For a long time, fish ammonia poisoning has been thought to be related to oxidative damage, such as in hybrid grouper *Epinephelus lanceolatus* ♂ × *Epinephelus fuscoguttatus* ♀ (Kim et al., 2020), flounder *Paralichthys olivaceus* (Cui et al., 2020), and golden pompano (Liu et al., 2021). It has been confirmed in mammals that stress causes overactivation of *N*-methyl-d-aspartate glutamate receptors in neurons, resulting in the production of a large number of reactive oxygen species (ROS), which is the main cause of oxidative damage (Hermenegildo et al., 2000). In general, the scavenging of ROS in fish depends on the activation of antioxidant enzymes (including SOD, CAT, and GPx), which are regulated by related genes expression (Trenzado et al., 2009). In this study, the *cu/zn sod*, *cat*, *gpx*, and *gr* expression were gradually up-regulating as ammonia levels increased, but the serum SOD, CAT, and GPx activities did not increase as expected. We speculated that this might be related to the excessive accumulation of MDA, because serum MDA contents continued to increase throughout the experiment. MDA can cross-link with the nucleophilic groups of proteins, nucleic acids, and amino phospholipids, leading to cytotoxicity and protein denaturation (Trenzado et al., 2009).

Previous studies have confirmed that the overproduction of ROS will further promote the release of proinflammatory cytokines (TNF, IL, and TGF) and induce apoptosis and even necrosis (Koca et al., 2008; Malaguarnera et al., 2009). TNFα is classified as a proinflammatory mediator that induces cell death by playing an initiating role in hepatocyte apoptosis; IL 1 stimulates T cell activation and promotes B cell proliferation and antibody secretion; IL 8 can recruit and activate macrophages and neutrophils, remove cell debris, and invade microorganisms (Budhu and Wang, 2006; Dhanasekaran and Reddy, 2008; Yin et al., 2014). In this study, the contents of serum proinflammatory cytokines (TNF, IL 1, and IL 8) were analyzed by the ELISA kit, and the *tnf ɑ*, *il 1*, and *il 8* expression levels were also analyzed. The results showed that they all increased with increasing ammonia levels, but we observed a decrease in inflammation with the dietary 0.05% NCG intake, which means that NCG can reduce the negative effects of inflammation.

During the bacterial challenge, ammonia stress resulted in increased CM while decreasing serum AT, LYZ, CH50, Ig, RB, and PI values. In higher animals, dietary NCG has a regulatory effect on their immune function. A dietary supplementation of 50 mg/kg NCG can improve the intestinal mucosal immune response of *Escherichia coli*-challenged piglets (Zhang et al., 2013). Moreover, dietary NCG supplementation has been found to enhance the immunity of *PRRSV*-infected sows (Yang et al., 2011). This study found that a dietary 0.05% NCG supplementation significantly reduced the CM of yellow catfish and significantly increased the serum immune response (AT, LYZ, Ig, and RB). So far, there have been few studies on the effects of dietary NCG on fish immunity and disease resistance; we speculate that nitric oxide may be involved. As an immunomodulatory molecule, nitric oxide is involved in regulation of T-lymphocyte proliferation, antibody immune response, and natural killer cells activity (Ye and Tian, 2010). As discussed earlier in this study, nitric oxide produced by nNOS can affect the brain function, but when produced by iNOS, it is involved in immune regulation. Based on the results of the correlation analysis between the nitric oxide synthase activity and ammonia metabolism enzyme activity, we propose: 1) when ammonia stress occurred, iNOS was positively correlated with ASS, ASL, and OTC activities (Figures 3B, C), which suggests that improving the urea cycle efficiency may effectively alleviate the negative effects of ammonia stress on the immunity of yellow catfish; 2) compared with the 0.00% NCG group (Figure 3D), iNOS in the 0.05% NCG group was only positively correlated with ASS activity (Figure 3E), which suggests that ASS may be another target of NCG to activate the urea cycle, though more evidence is needed.

## 5 Conclusion

When ammonia stress occurs, dietary NCG supplementation can improve the growth, hematological index, oxidation resistance status, immune response, and disease resistance of yellow catfish, and it can thus improve their ammonia tolerance. This study hypothesized that ASS may be another target of NCG to activate the urea cycle.

## Data Availability

The original contributions presented in the study are included in the article/supplementary material, further inquiries can be directed to the corresponding author.
